# Evidence for Existence of Multiple Functional Human Small RNAs Derived from Transcripts of Protein-Coding Genes

**DOI:** 10.3390/ijms24044163

**Published:** 2023-02-19

**Authors:** Fan Gao, Fang Wang, Huifen Cao, Yue Chen, Yong Diao, Philipp Kapranov

**Affiliations:** Institute of Genomics, School of Medicine, Huaqiao University, 668 Jimei Road, Xiamen 361021, China

**Keywords:** small RNA, short RNA, noncoding RNA, RNA dark matter, high-throughput phenotypic assay

## Abstract

The human genome encodes a multitude of different noncoding transcripts that have been traditionally separated on the basis of their lengths into long (>200 nt) or small (<200 nt) noncoding RNAs. The functions, mechanisms of action, and biological relevance of the vast majority of both long and short noncoding transcripts remain unknown. However, according to the functional understanding of the known classes of long and small noncoding RNAs (sncRNAs) that have been shown to play crucial roles in multiple biological processes, it is generally assumed that many unannotated long and small transcripts participate in important cellular functions as well. Nevertheless, direct evidence of functionality is lacking for most noncoding transcripts, especially for sncRNAs that are often dismissed as stable degradation products of longer RNAs. Here, we developed a high-throughput assay to test the functionality of sncRNAs by overexpressing them in human cells. Surprisingly, we found that a significant fraction (>40%) of unannotated sncRNAs appear to have biological relevance. Furthermore, contrary to the expectation, the potentially functional transcripts are not highly abundant and can be derived from protein-coding mRNAs. These results strongly suggest that the small noncoding transcriptome can harbor multiple functional transcripts that warrant future studies.

## 1. Introduction

The human genome is pervasively transcribed to generate multiple different classes of noncoding transcripts [1,2,3,4,5,6,7,8,9], the so called “RNA dark matter” [10,11]. Such transcripts are separated on the basis of their length into long (>200 nt) and small (<200 nt) noncoding RNAs [7], with the former representing the majority of transcriptional output of a mammalian genome by relative mass [12] or sequence complexity [8]. Therefore, due to their widespread abundance and potential importance as a novel type of regulatory molecules [13,14], the discovery, characterization, and functional annotation of long noncoding (lnc) RNAs have attracted a vast amount of scientific interest in the past two decades [9,15]. Furthermore, while the debate about their functionality persists [16], potential models of their mechanisms of function have been generated, and a number of lncRNAs have been characterized in great detail [9,17,18].

On the other hand, the human sncRNA transcriptome is also quite abundant and diverse; it consists of not only several known and well-characterized classes of RNAs, but also, as revealed by multiple reports, various novel classes of transcripts [7,8,19,20,21,22,23,24,25,26,27]. In addition to the structural sncRNAs such as ribosomal (r), transfer (t), and small nuclear (sn) RNAs, several other classes of sncRNAs with well-understood functions and mechanisms of action, such as small interfering (si) [28], micro (mi) [29,30], PIWI-interacting (pi) [31], and small nucleolar (sno) [32] RNAs, exist and contribute thousands of different annotated RNAs [33,34,35,36] to the human small RNA transcriptome.

Nevertheless, the whole human sncRNA transcriptome is far more complex than can be explained by the annotated small RNAs. For example, a study using high-density tiling arrays found 447,406 sncRNAs in two human cell lines, the vast majority of which mapped outside of known sncRNAs such as miRNAs, snoRNAs, and others, thus representing novel, unannotated sncRNA species [7]. The study also found significant enrichment of sncRNAs around transcriptional start sites (TSSs) and termination sites (TTSs) of annotated genes that the authors assigned to the classes of “promoter-associated sRNAs” (PASRs) and “termini-associated sRNAs” (TASRs), respectively [7]. The association of mammalian sncRNAs and gene boundaries, in both sense and antisense directions, as well as with splice sites, has also been found by several subsequent reports based on next-generation sequencing (NGS) of small RNA populations in multiple species [8,19,20,21,22,23,24,25,26,27], resulting in the discovery of tiny RNAs [19,20], TSSa-RNAs [21], aTASRs [22], and TTSa-RNAs [23]. Notably, using NGS, the ENCODE consortium has found >150,000 human unannotated sncRNAs, of which ~40% represented PASRs and TASRs [8]. Strikingly, sncRNAs have been found to associate with sites of double-strand DNA breaks in different organisms, leading to a tantalizing possibility that such transcripts can guide the DNA repair machinery to the sites of DNA damage [37,38,39].

It is generally assumed that the majority of novel sncRNAs are produced by post-transcriptional cleavage of longer transcripts [40], similar to the mode of biogenesis of known classes of sncRNAs such as miRNAs [29]. On one hand, sncRNAs could be produced from much longer precursor transcripts, as illustrated by multiple miRNAs generated from introns of annotated genes via cleavage by two type III ribonucleases, Drosha and Dicer [29]. On the other hand, sncRNAs can also be generated by cleavage of other small RNAs, as illustrated by the discovery of stable small fragments of tRNAs [41] and other structural RNAs [40]. Overall, it appears that post-transcriptional cleavage is a wide-spread phenomenon that can explain the generation of multiple long and small RNA species [42,43,44,45], as reviewed in [40]. In fact, the genomic overlap between unannotated long and small RNAs has led to a hypothesis that a significant fraction of the lncRNA transcriptome functions as a precursor to sncRNAs [7].

Nevertheless, despite significant progress made in mapping the short RNA transcriptome, as well as the evidence of functionality of some novel sncRNAs [25,46], the question about the biological relevance of the vast majority of unannotated sncRNA species remains mostly unanswered. While the same issue also fuels the debate about the functionality of lncRNAs, this issue is even more pronounced in the case of sncRNAs since, unlike lncRNAs which are often produced by dedicated promoters and resemble *bona fide* genes [15], the unannotated sncRNAs can simply represent stable degradation products of longer transcripts that have no function. Therefore, in this study, we investigated whether, in principle, unannotated sncRNA can be functional by assessing phenotypic effects of their overexpression in human cancer cells. Strikingly, we found that a significant fraction of the tested sncRNAs, including small RNAs derived from coding regions of exons of mRNAs, appeared to have phenotypic consequences in our assay after overexpression, suggesting that the unannotated sncRNA transcriptome might indeed harbor novel and functional RNA species that are not mere degradational byproducts of longer transcripts.

## 2. Results

### 2.1. Properties of sncRNA Transcriptome in a Human Cancer Cell Line

Currently, it is not possible to predict whether an unannotated sncRNA that does not belong to a known class of short RNAs is biologically relevant or not. Therefore, in this work, we investigated the biological relevance of unannotated sncRNAs on the basis of two different features which would normally be assumed to associate with functional sncRNAs. One such feature is based on the assumption that at least some biologically relevant sncRNAs should function via specific motifs embedded in their sequences, as exemplified by the base-pairing-mediated interactions between miRNAs and their targets [47]. Therefore, precise processing of such sncRNAs on both 5′ and 3′ ends by dedicated cellular enzymes—as in the case of mature miRNAs [29]—would be critical for their functions. Thus, unannotated sncRNAs whose boundaries are conserved, i.e., detected precisely, in different batches of cells from the same cell type that were grown by independent groups would be more likely to represent true biologically relevant sncRNAs. The second feature is transcript abundance since sncRNAs that belong to annotated classes are known to be highly abundant in the cells [8].

**Figure 1:**
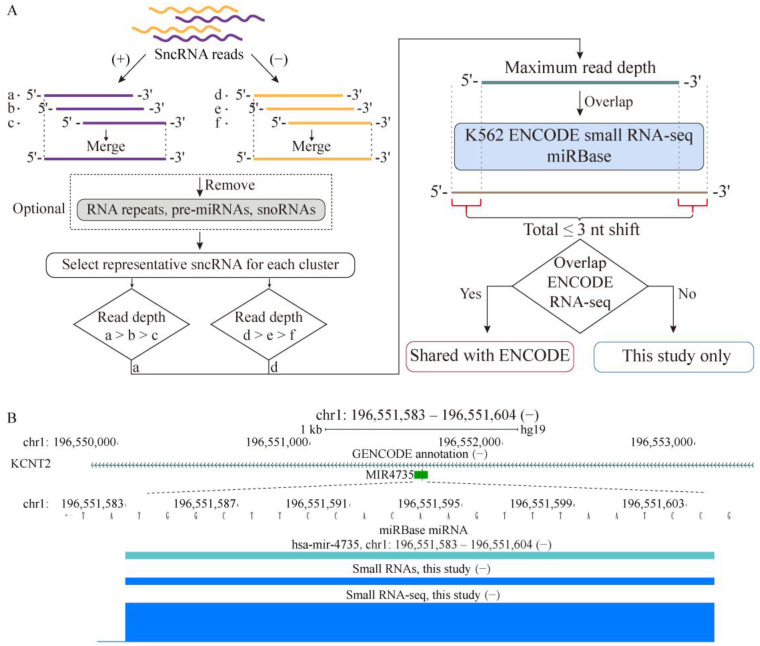
Profiling sncRNA transcriptome in a human cancer cell line. (**A**) Schematics illustrating the discovery and characterization of sncRNA transcriptome performed in this work. Small RNA-seq analyses from three independent batches of K562 cells were combined, and reads overlapping on the same strand were merged into clusters. For analysis of unannotated sncRNAs only, the clusters were filtered to remove those that overlap with the RNA family of repeats and annotated mi- and snoRNAs. Then, for each cluster, a representative sncRNA with unique 5′/3′ coordinates and maximum read depth was selected. The same procedure was also applied to the ENCODE small RNA-seq data without the optional filtering step. An sncRNA whose 5′ or 3′ coordinates mapped within 3 bp of an miRBase miRNA was considered as having precise overlap with that miRNA. An unannotated sncRNA detected in this study that overlapped an ENCODE sncRNA using the same overlap criterion was considered as shared with the ENCODE dataset. (**B**) Example of precise detection of a mature miRNA in our small RNA-seq pipeline. Shown are the locations and genomic coordinates of the mature hsa-mir-4735 (based on miRBase) and sncRNAs generated by our pipeline from the K562 small RNA-seq data produced in this study. In addition, the normalized RNA-seq signal from this study is shown. Genomic coordinates of the sncRNA detected in this study are shown above the panel.

As the first step, we investigated the overlap between two sncRNA profiles from the same cell line generated at different times by different groups. We performed RNA-seq profiling of a small RNA population, specifically targeting the 20–40 nt range, in the human leukemia K562 cell line and compared it with the sncRNA profile from the same cell line generated previously by the ENCODE consortium [8]. The rationale behind choosing this size range was twofold: (1) limiting the complexity of the profiled sncRNA transcriptome in order to detect relatively low abundant unannotated sncRNAs, and (2) the existence of at least three well-characterized classes of sncRNAs, si-, mi-, and piRNAs, which are known to fall into this size range [28,29,31], making it possible that other classes of functional sncRNAs can also be found in this range. We generated a total of ~62 M NGS reads from three independent batches of K562 cells. As shown in the Supplementary Figure S1, the genomic distribution of reads across the three biological replicates was quite similar; therefore, for the analyses below, we combined the NGS reads from the three replicates. We then generated 2,633,758 unique sncRNA species defined by the following two-step procedure: (1) small RNA-seq reads overlapping on the same strand were merged; (2) for each merged cluster, reads with unique 5′ and 3′ ends that had maximum depth were selected to represent that cluster (Figure 1a; see Section 4). We also processed ~102 M reads from the ENCODE whole-cell K562 data using the same procedure to generate 1,200,457 unique ENCODE K562 sncRNAs.

As illustrated in Figure 1b, the boundaries of mature miRNAs detected in our small RNA-seq data were quite consistent with those annotated in miRBase or detected by the ENCODE dataset. To allow for the sequencing and alignment errors, we allowed a total shift of three bases in both 5′/3′ boundaries of matching sncRNAs to consider them as having precise overlap (Figure 1a). We could detect 1565 mature miRNAs annotated in the miRBase database [33] in the K562 cell line with any overlap, of which 1325/1565 or 85% had precise overlap (Figure 1a). Of the 1565 miRNAs, 936 (60%) could also be detected in the K562 ENCODE unique sncRNA dataset, of which the majority (~79%, 737/936) had precise overlap (Figure 1a; see Section 4). Thus, at least according to the well-characterized class of miRNAs, as expected, most of the functional sncRNAs had precise boundaries between the different datasets.

**Figure 2:**
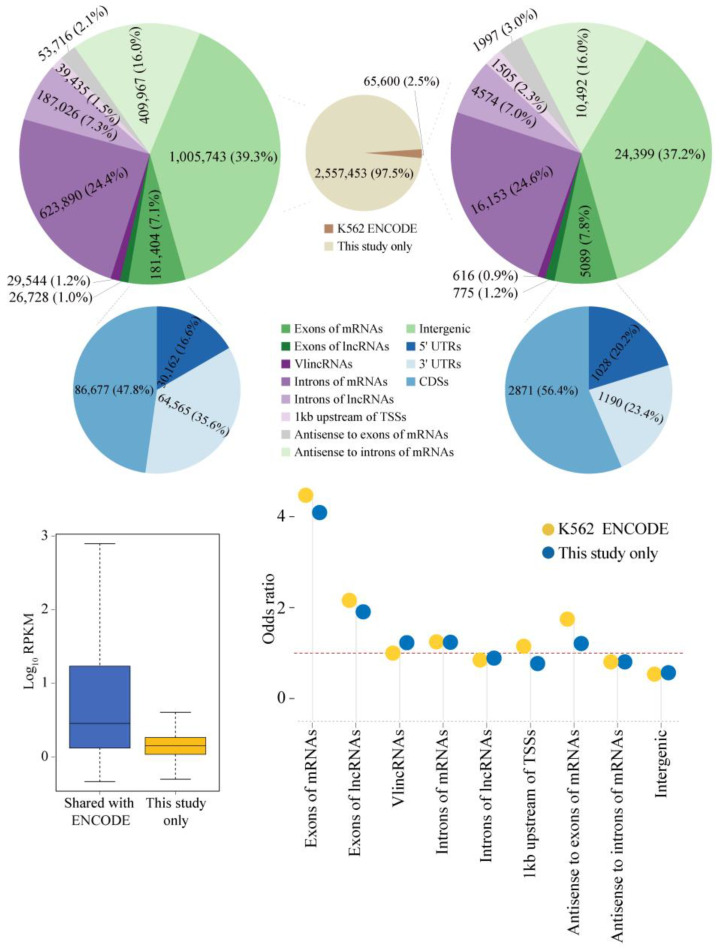
Properties of unannotated sncRNA transcriptome in a human cancer cell line. Top: The middle pie chart shows the fractions of the sncRNAs shared with ENCODE and those found only in this study. The left and right pie charts show the numbers and fractions of unannotated sncRNAs mapping to the various indicated genomic features for respectively this-study-only and shared K562 sncRNAs, respectively. Bottom left: Box plots of expression levels (log_10_ RPKM, Y-axis) of shared (left) and this-study-only (right) K562 sncRNAs. Bottom right: Odds ratios (Y-axes) of enrichment of this-study-only and shared K562 sncRNAs in the different types of genomic elements (X-axes). The red dashed horizontal lines represent odds ratios of 1, corresponding to no enrichment.

As the next step, we investigated the properties of unannotated sncRNA transcriptome by filtering our K562 sncRNA-seq data to remove transcripts overlapping known classes of sncRNAs (see Section 4) to generate 2,623,053 unique unannotated sncRNA species. Consistent with the initial targeted range of 20–40 nt chosen during the library construction, the actual lengths of sncRNAs ranged from 10 to 42 nt with the median length of 15 nt according to the analysis of the NGS reads. The small shift toward the lower size range was likely caused by preferential amplification of shorter sequences during the PCR steps of the library construction protocol. Of the 2,623,053 unannotated sncRNAs, 2.5% (65,600) had precise overlap with the unique ENCODE sncRNAs (Figure 2; see Section 4). The sncRNAs shared with the ENCODE dataset had significantly higher abundance (*p*-value < 2.20 × 10^−16^, two-sided Wilcoxon rank sum test) compared to the sncRNAs detected only in this study (Figure 2).

**Figure 3:**
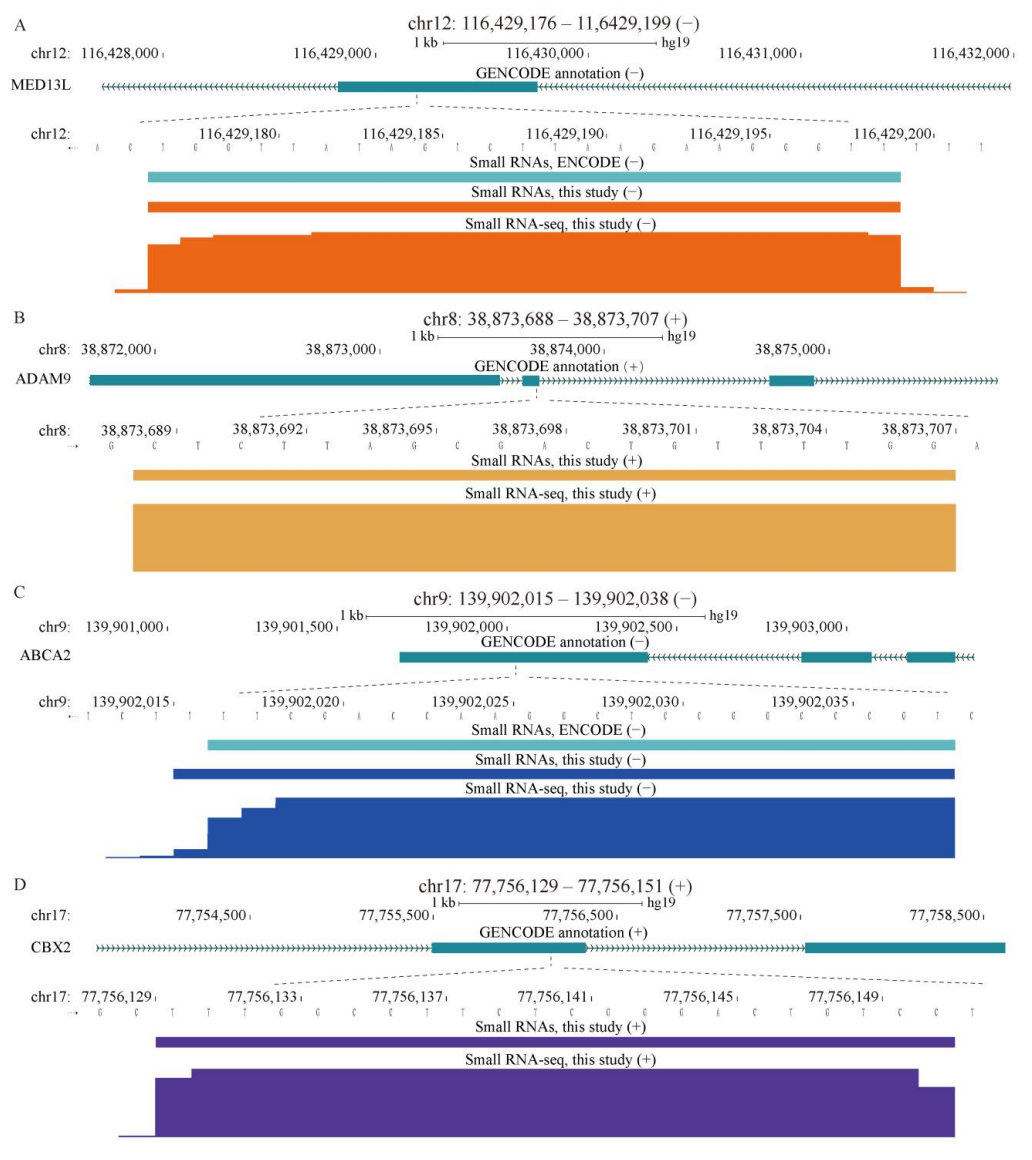
Examples of sncRNAs mapping to exons of protein-coding genes. SncRNAs mapping to the CDS (**A**,**B**) and 3′ UTR (**C**,**D**) regions are shown for the sncRNAs that are either shared with ENCODE (**A**,**C**) or unique to this study (**B**,**D**). Locations of the sncRNAs generated by our pipeline from the K562 small RNA-seq data produced in this study (**A**–**D**) and by ENCODE (**A**,**C**) are shown. (**A**–**D**) The normalized RNA-seq signal from this study is shown. Genomic coordinates of the sncRNAs detected in this study are shown above the panels.

Analysis of association of the unannotated sncRNAs with multiple genomic features revealed a very strong association with exons of protein coding genes and, to a lesser extent, with exons of lncRNAs, both in the same strand polarity (Figure 2), as illustrated by specific examples shown in Figure 3a–d. This association could be found in sncRNAs detected only in our K562 data, but it was stronger in the sncRNAs shared with the ENCODE dataset (Figure 2). The odds ratios of overlap with exons of mRNAs for the K562 sncRNAs detected only in this study or shared with ENCODE were 4.09 and 4.47, respectively, while the corresponding ratios for the overlap with exons of lncRNAs were 1.91 and 2.16 (Figure 2 and Supplementary Table S1). The overlap was significant with all portions of mRNAs, i.e., 5′ and 3′ UTRs, as well as the coding regions (CDSs, Figure 2 and Figure 3a,b). However, the strongest overlap was observed with CDSs with the odds ratio of 7.61 for the shared K562 sncRNAs, which was higher than the corresponding value (5.90) for sncRNAs found only in this study (Figure 2 and Supplementary Table S1). Overall, of all exon-associated sncRNAs, 47.8% and 56.4% mapped to CDSs either for this-study-only or for shared groups of transcripts (Figure 2, Supplementary Table S1).

The overlap with 3′ UTRs was also quite strong with the odds ratios for this-study-only and shared sncRNAs, being 6.45 and 4.64, respectively (Figure 2 and Figure 3c,d, and Supplementary Table S1). The corresponding odds ratios for 5′ UTRs were much lower, albeit still indicative of enrichment (1.29 and 1.72, respectively; Figure 2 and Supplementary Table S1), consistent with the existence of PASRs and other TSS-associated classes of sncRNAs. Interestingly, we could also detect relatively weak, but significant (odds ratios of 1.21 and 1.75) enrichment of snRNAs on the opposite strand (antisense) to exons of mRNAs (Figure 2 and Supplementary Table S1).

**Figure 4:**
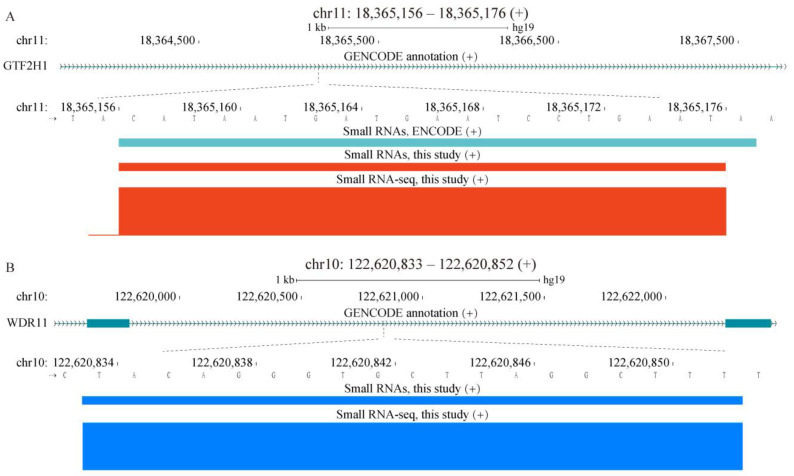
Examples of sncRNAs mapping to introns of protein-coding genes. Locations of the sncRNAs generated by our pipeline from the K562 small RNA-seq data produced in this study (**A**,**B**) and by ENCODE (**A**) are shown for the shared (**A**) or this-study-only (**B**) sncRNAs mapping to introns. (**A**,**B**) The normalized RNA-seq signal from this study is also shown. Genomic coordinates of the sncRNAs detected in this study are shown above the panels.

Nevertheless, only 7.1% or 7.8% of sncRNAs (this-study-only or shared with ENCODE, respectively) overlapped exons of mRNAs on the same strand with additional 1% or 1.2% mapping to exons of lncRNAs (Figure 2 and Supplementary Table S1). On the other hand, introns of mRNAs represented an additional significant reservoir of sncRNAs with 24.4% and 24.6% of this-study-only and shared sncRNAs mapping to the same strands of these regions (Figure 2 and Figure 4a,b, and Supplementary Table S1). Furthermore, sncRNAs exhibited weak but significant enrichment in the mRNA introns with the odds ratios of 1.24 and 1.25 (Figure 2 and Supplementary Table S1). Altogether, these results showed that, even though a majority of sncRNAs were specific to our K562 RNA-seq data, a large number (65,600) of sncRNAs had conserved boundaries with the ENCODE K562 data, which was consistent with potential functionality of at least some of these RNA species. Moreover, while such sncRNA species could be derived from different genomic elements, we found striking enrichment in the sequences otherwise used to encode exons of mRNAs.

### 2.2. Establishing Overexpression System for Unannotated sncRNAs

While consistent with functionality of an sncRNA, conservation of its boundaries in different batches of the same cell type combined with the higher abundance of such sncRNA species is not proof that the sncRNA can have an impact on cellular physiology. Assessment of phenotypic consequences following perturbation of expression levels of a transcript is widely used as evidence of functionality of that RNA species. One way to achieve this is to deplete the target RNA using antisense oligos, RNAi, or the CRISPR suite of genome-editing or RNA targeting tools [48]. However, in this case, such an approach would have at least two major problems. First, as shown above, many sncRNAs overlapped with exons or introns of other transcripts on the same strands; therefore, targeting the small RNAs would also deplete the long RNA species, causing ambiguities in interpreting the results of any phenotypic studies. Second, existing RNA depletion methods are known to have strong off-target effects that would also cause problems in interpreting the effects of such assays [48].

**Figure 5:**
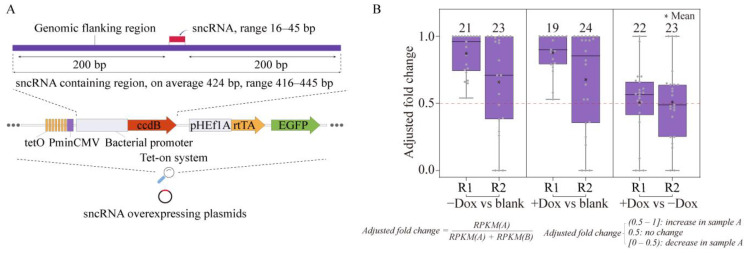
Design and validation of the sncRNA overexpression strategy. (**A**) Strategy of sncRNA overexpression in a lentiviral vector. A genomic region containing the target sncRNA plus ~200 bp of flanking DNA on each site is cloned in place of the suicide ccdB cassette downstream from a strong Dox-inducible RNA Pol2 promoter. (**B**) Validation of the strategy by transient overexpression of selected unannotated sncRNAs in HEK293 cells. For each biological replicate (R1 and R2), box plots of adjusted fold changes (Y-axis) of the sncRNAs are shown for subsequent comparisons. Left and center: Cells transfected with the pool of overexpressing vectors vs. cells transfected with the empty vector (the blank control). The cells were either treated (middle) or not treated (left) with Dox, while the blank control cells were not treated with Dox. Right: +Dox vs. −Dox treatments of cells transfected with the pools of overexpressing vectors. The adjusted fold change can range from 0 to 1, with 0.5 marked by the red dashed line representing no change. Values shifted toward 1 in the left and middle plots indicate overexpression compared to the blank controls. The number of sncRNAs which had RPKM >0 in the corresponding sample and used to generate each box plot is shown above.

Therefore, we explored overexpression as a strategy to perturb the expression of specific sncRNAs. However, overexpression by transfecting synthetic RNA species into cells has been shown to cause nonspecific effects by generating aberrant RNA species caused by accumulation of supraphysiological amounts of transfected RNA molecules inside the cells [49]. Therefore, to avoid this issue, we chose to overexpress sncRNAs by expressing longer transcripts that contained the corresponding sncRNAs flanked by short (200 bases on average) genomic sequences at both sides, which should avoid such issues [49] (Figure 5a; see Section 4). Assuming that many unannotated sncRNAs would be generated as products of cleavage from longer precursors, it would be expected that sequences flanking sncRNAs should contain sequence/structure motifs required for proper biogenesis of such small transcripts by analogy with the well-understood principles of biogenesis of miRNAs [29,50]. Furthermore, similar overexpression strategies have been used successfully to overexpress specific miRNAs [51,52].

To test whether such strategy could indeed result in overexpression of specific unannotated sncRNAs, we chose 34 such transcripts from the unannotated K562 sncRNAs. The corresponding genomic sequences were inserted under the control of a tetracycline-inducible promoter in a lentiviral vector and used to transduce HEK293 cells as a pool (Figure 5a; see Section 4). The transduced cells were then cultured for 24 h either with or without doxycycline (Dox) to induce the expression of the sncRNA-containing transcripts. Cells transduced with an “empty” vector, without the sncRNA-containing regions, were used a “blank” control and were grown without Dox. The cells containing stable integrations of the viral genomes following transduction either with the library of sncRNA-containing regions or empty vector (Figure 5a) were then selected using cell sorting according to the expression of GFP encoded by the vector and subjected to sncRNA profiling using RNAseq.

Interestingly, we could observe a significant increase in the expression levels of the sncRNAs in cells transduced with the vectors containing the sncRNA-containing regions compared to the blank controls in two independent bioreplicates (Figure 5b). The increase was apparent in cells that were either grown with or without Dox (+Dox or −Dox, respectively, Figure 5b). In fact, of 34 tested sncRNAs, 27 could be detected by RNA-seq in at least one sample, of which 20 (74%) were found to be induced in at least two of the four samples transfected with the sncRNA overexpressing vectors (two bioreplicates of each +Dox and −Dox cells, Supplementary Table S2). These results suggest that this strategy could indeed result in the overexpression of unannotated sncRNAs. The increase in expression found in the −Dox cells was not unexpected given the well-known leaky expression of transgenes in the tetracycline-inducible expression systems [53,54,55]. On the other hand, surprisingly, the average level of induction in the +Dox compared to the −Dox cells was rather small (Figure 5b). One possible explanation of this phenomenon could be detrimental effect of Dox-induced high levels of expression of sncRNAs on cell growth and survival, leading to loss of cells with high expression of sncRNAs. This would be consistent with biological relevance of these small RNAs; therefore, we further investigated this possibility, as described below.

### 2.3. Phenotypic Analysis of Selected sncRNAs

**Figure 6:**
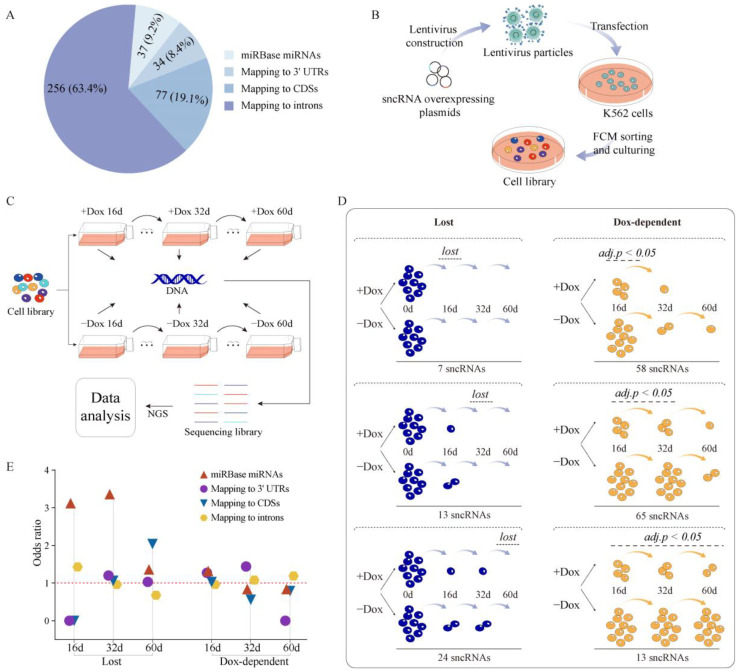
High-throughput phenotypic screen of selected sncRNAs. (**A**) Composition of the selected 404 sncRNAs chosen for the phenotypic screen. (**B**) Schematics of the library preparation for the high-throughput phenotypic screen. A pool of the 404 lentiviral sncRNA inducible overexpression plasmids was used to generate lentiviral particles, which were then used to transduce K562 cells under the conditions that favor single viral integration event per cell. Two million stably transduced cells were selected by flow cytometry (FCM) and expanded to generate the starting cell library for the phenotypic screen. (**C**) Schematics of the growth survival assay. The starting cell library was subjected to culture in presence or absence of Dox for 60 days. Genomic DNA was isolated at days 16, 32, and 60 and used to quantify the sequences corresponding to each of the 404 sncRNA overexpression regions by NGS. (**D**) Results of the phenotypic screens. The functional sncRNAs were identified on the basis of depletion of the corresponding overexpression regions and could be subdivided into two categories. The “lost” sncRNAs were lost in both +Dox and −Dox samples after 16, 32, or 60 days of culture compared to the day 0 library. The “Dox-dependent” sncRNAs exhibited statistically significant loss in the +Dox samples compared to –Dox ones the after 16, 32, or 60 days of culture as estimated by the adjusted fold change, adjusted p-value, and average levels compared to the day 0 cultures (see Section 4 for details). (**E**) Odds ratios (Y-axes) of enrichment of the four groups of the original 404 sncRNAs—the positive control miRNAs and the unannotated sncRNAs that overlapped with introns, CDSs, or 3′ UTRs—among the functional sncRNAs in the “lost” and “Dox-dependent” categories at the different timepoints (X-axes). The red dashed horizontal line represents the odds ratio of 1 corresponding to no enrichment.

To perform a deeper analysis of the potential inhibitory effect of the overexpression of unannotated sncRNAs on cellular growth and survival, we used the same strategy to generate an overexpression library of 358 sncRNAs detected in K562 and containing 328 unannotated sncRNAs, as well as 30 miRBase miRNAs as positive controls. The 328 sncRNAs consisted of 198 sncRNAs shared with the ENCODE K562 dataset and 130 sncRNAs detected only in this study. We also included additional 39 unannotated sncRNAs and seven miRNAs that were not detected in our K562 RNA-seq data but detected in the ENCODE RNA-seq data (either in K562 or in other cell lines) to test whether sncRNAs expressed in other cell lines or in the same cell line but under different growth conditions could have a function in that cell line. Overall, we tested a total of 404 sncRNAs in the overexpression assay (Figure 6a, Supplementary Table S3). To limit the scope of this pilot study, we focused solely on unannotated sncRNAs that could be derived by cleavage of transcripts originating from the annotated protein-coding gene loci: the selected sncRNAs mapped within boundaries of protein-coding genes and on the same strands as the corresponding genes. Since most miRNAs that are found within genes reside inside introns, we hypothesized that functional sncRNAs could also be enriched in introns. Therefore, out of the 367 unannotated sncRNAs chosen for functional work, 256 (~70%) were located in introns. However, because of the striking overlap between sncRNAs and exons of mRNAs, we also included 77 sncRNAs derived from CDSs and 34 from 3′ UTRs (Figure 6a). At this stage, we decided to avoid TSS-associated sncRNAs since at least some of them could represent products of stalled RNA Pol 2 transcription rather than cleavage [26,56]; thus, their overexpression would require a different strategy. A pool of lentiviral vectors representing the 404 sncRNAs was used to transfect K562 cells (Figure 6b) under the conditions that favor a single viral integration event per cell, essentially as described previously [57] (see Section 4). Two million stably transduced cells were selected by flow cytometry and further expanded to generate the sncRNA overexpression cell library (Figure 6b; see Section 4). The sncRNA overexpressing regions were then PCR amplified from the genomic DNA with common primers and subjected to NGS ensure the quality of the library that had the coverage of 100% and evenness of 11.3 with the latter calculated as the ratio of counts of the region in the 90th percentile to those in the 10th.

The overexpression library was then cultured with (+Dox) or without (−Dox) in three parallel replicates (Figure 6c). To ensure proper coverage, each time course experiment started with one million cells, thus equaling >2000 cells per each sncRNA. After 16 and 32 days, aliquots of at least one million cells were taken for genomic DNA isolation from each sample, while the remaining cells were allowed to continue in culture until day 60, at which point genomic DNA was also isolated and the time course terminated (Figure 6c). If overexpression of a specific sncRNA is indeed detrimental to cell growth and survival, then it would be expected that cells containing insertions of the overexpression cassette for such sncRNA get depleted from the population. The depletion should then be reflected in the loss of the corresponding sncRNA sequences from the pool of genomic DNA isolated from the cells which could be quantified by NGS (see Section 4). Therefore, to identify sncRNAs that affected cell growth and survival, the genomic regions containing the sncRNAs were then PCR amplified from each genomic DNA sample using a pair of common primers and subjected to NGS to quantify presence of each sncRNA (see Section 4).

The resulting normalized NGS read counts for each sncRNA (Supplementary Table S4) were compared in two different ways (Figure 6d). First, since phenotypic effect of an sncRNA could be observed even without Dox due to leaky expression, the counts of each sncRNA in both +Dox and −Dox samples were compared to the counts in the original library prior to the time course of culture. Second, the counts in the +Dox samples were compared to those in −Dox ones. Using this approach, we identified multiple sncRNAs for which the overexpression was detrimental to cells; however, interestingly, we found no sncRNAs for which overexpression promoted cell growth. In other words, we could detect only loss of sncRNAs, but not gain, during the time course of culture (Figure 6d). As expected, some sncRNAs were lost in both +Dox and −Dox samples. After 16 days of culture, seven sncRNAs were totally lost in both +Dox and −Dox samples (Figure 6d, Supplementary Tables S4 and S5). Additional 37 sncRNAs dropped to very low levels (on average, 18% of the levels in the original library before culture) in both +Dox and −Dox samples after 16 days of culture, and then 13 and 24 were totally lost in both types of samples after 32 and 60 days of culture, respectively (Figure 6d, and Supplementary Tables S4 and S5).

On the other hand, many sncRNAs exhibited Dox-dependent depletion at various timepoints of culture. For example, 58 sncRNAs showed statistically significant loss after 16 days of culture in the +Dox compared to the −Dox samples followed by loss in both (+ or −Dox) samples on days 32 and 60 (Figure 6d, and Supplementary Tables S4 and S5). Likewise, an additional 65 sncRNAs exhibited statistically significant Dox-dependent loss after 32 days of culture followed by loss in both (+ or −Dox) samples after 60 days (Figure 6d, and Supplementary Tables S4 and S5). An additional 13 sncRNAs showed statistically significant Dox-dependent loss after 60 days of culture (Figure 6d, and Supplementary Tables S4 and S5). Overall, out of the 404 sncRNAs tested, 180 or 44.6% showed evidence of detrimental effect on cells in this overexpression assay. Furthermore, for 136/180 or ~75.6% such sncRNAs, this effect was dependent on Dox, which is consistent with RNA being the mediator of the observed phenotypic effect.

Overall, as expected, a higher fraction (22/37 or 59.5%) of the positive control set, the miRBase miRNAs, was found to have effect in our assay across all samples compared to that of unannotated sncRNAs (158/367 or 43.1%; Figure 6d and Supplementary Table S5). Furthermore, sncRNAs depleted early in the time course would be expected to have stronger deleterious effects on cells. Moreover, it is reasonable to assume that the deleterious effects of sncRNAs depleted in the −Dox samples was caused by their background level of expression due to promoter leakiness; thus, such sncRNAs should represent transcript with stronger phenotypic effects. Therefore, we tested whether sncRNAs of different categories used in this study had different enrichment at different timepoints and also in both (+ or −Dox) samples or only in the +Dox samples. Interestingly, the miRBase miRNAs were strongly enriched in the sncRNAs depleted after 16 and 32 days of culture in both + or −Dox samples and after 16 days in the Dox-dependent fashion (Figure 6e and Supplementary Table S5). These results are consistent with well-known physiological functions and mechanisms of action of this class of sncRNAs (see Section 3) and provide further validation of our assay.

### 2.4. Properties of Unannotated sncRNAs Positive in the Phenotypic Screen

**Figure 7:**
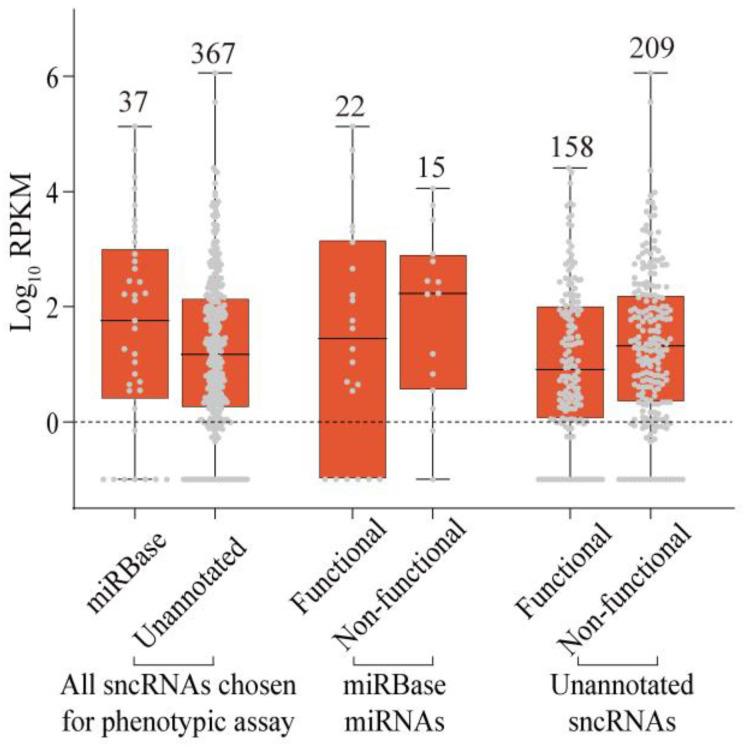
Comparison of expression levels of different groups of sncRNAs used in the phenotypic screen. Box plots of expression (log_10_ of RPKM, Y-axes) of the 404 sncRNAs chosen for the functional assay and stratified by representing the miRNAs positive controls vs. the unannotated sncRNAs and functional vs. nonfunctional status in the assay. The number of sncRNAs used to generate each box plot is shown above. Grey dots represent individual data points. The dashed line represents the boundary of low sncRNA abundance — RPKM of 1 (0 on the log-scale).

The unannotated sncRNAs positive in the phenotypic assay were approximately evenly distributed across all genomic elements tested in this study—3′ UTRs, CDSs, and introns—with the corresponding fractions of 50% (17/34), 40.3% (31/77), and 43.0% (110/256; Supplementary Table S5). Strikingly, and contrary to the original expectations, the fraction of sncRNAs positive in the phenotypic screen was lower among the sncRNAs shared with the ENCODE dataset compared to those found only in this study: 38.8% (83/214) vs. 49% (75/153). We hypothesized that this might be explained by the abundance, since sncRNAs shared with the ENCODE dataset had a tendency to be more abundant than those found only in this study (Figure 2). Indeed, we found that transcript abundances of the sncRNAs that had phenotypic effects on cells were lower than those of sncRNAs overexpression of which had no consequences to cell growth and survival (Figure 7 and Supplementary Table S6). Interestingly, this was true for both miRNAs and unannotated sncRNAs. The median RPKM values for miRNAs that were positive or negative in our screen were 30.2 and 168.9, respectively, while the corresponding values for the unannotated sncRNAs were 8.2 and 20.9 (Figure 7 and Supplementary Table S6). The lower abundance of the functional sncRNAs was statistically significant with a *p*-value of 0.02 (one-sided Wilcoxon rank sum test). Furthermore, the sncRNAs that were not detected in K562 but detected in other cell lines were more likely to be functional in our assay, as illustrated by 85.7% (6/7) and 56.4% (22/39) of miRNAs and unannotated sncRNAs, respectively (Supplementary Table S6). These observations contrasted with the fact that the expression levels of the 30 positive control miRNAs used in our assay were about an order of magnitude higher than those of the other 328 sncRNAs, with median RPKMs of 167.6 and 22.9, respectively (Figure 7). Thus, while a large fraction (43.1%) of unannotated sncRNAs had evidence of biological function in our assay, the features of these sncRNAs were different from what might have been expected *a priori* (see Section 3).

## 3. Discussion

This work represents a proof-of-principle study to show the physiological relevance of unannotated sncRNAs in a high-throughput assay using cell growth and survival as the readout phenotype. To our knowledge, this is the first study that attempted to develop and apply a high-throughput phenotypic assay to study the functionality of unannotated sncRNAs. There are two main arguments that support the validity of our assay. First, the Dox dependency of the observed phenotypic effect for the majority of sncRNAs strongly argues that the phenotypic effect was mediated by RNA. Second, the members of the annotated functional class of miRNAs had a higher fraction of positives in our assay compared with the unannotated sncRNAs.

Strikingly, we found that a significant fraction (43.1%) of all tested unannotated human sncRNAs had evidence of physiological relevance in this assay. The remaining sncRNAs either represented stable degradation products with no biological function or their functionality failed to be revealed due to the limitations of the overexpression assay (see below). It is important to emphasize that the sncRNAs tested in this work were not selected randomly; in fact, they were chosen as potential cleavage products of transcripts sharing the same boundaries and strands with annotated human genes—pre-mRNAs, mature miRNAs, or overlapping lncRNAs. However, considering that we found 1,110,998 sncRNAs located within gene boundaries in just one human cell line, our results would indicate that the total pool of functional human unannotated small transcriptome numbers in thousands of RNA species.

Unexpectedly, the sncRNAs that could be independently detected in multiple sncRNA profiling experiments were less enriched in functional sncRNA species compared to those found only in this study. The most likely explanation of this result is that the sncRNAs associated with the phenotype had a tendency to have lower abundance and, thus, were less likely to be detected in different RNA-seq datasets. It is important to emphasize that we observed this phenomenon for both unannotated sncRNAs and miRNAs. The biological relevance of low-abundance sncRNAs is contrary to the conventional logic that stipulated that higher abundance should correlate with biological relevance. However, there are theoretical scenarios where low sncRNA abundance is compatible with functionality. For example, expression of a functional sncRNA could be restricted to a specific subpopulation of K562 cells and appear to have low abundance in bulk RNA-seq analysis. It is conceivable that such functional sncRNAs whose expression has to be tightly controlled would be more likely uncovered in a phenotypic assay that is based on ectopic overexpression of sncRNAs cells, where they are normally not expressed.

Consistent with this notion, as shown by the study of Isakova et al. who performed single cell profiling of small and long transcriptomes of cultured mammalian cells, the expression of subset of miRNAs and many other sncRNAs strongly depended on the cell-cycle stage [58]. Furthermore, as shown by Liu et al., transcriptome profile heterogeneity of long RNAs among cultured cells is much higher than previously anticipated [59]. Overall, these results suggest that sncRNAs with relatively low abundance can still have biological function, and that abundance level, at least estimated on bulk cell transcriptomes, cannot be used as a universal predictor of the biological relevance of a transcript.

The potential existence of multitude of functional sncRNAs poses an obvious question of possible mechanisms of function of the unannotated sncRNAs. Interestingly, recent studies have uncovered evidence supporting existence of multiple novel mechanisms of sncRNA function which at least partially rely on the components of already characterized sncRNA pathways. For example, the study of Cass et al. identified 398 mammalian sncRNAs that could induce endonuclease-mediated cleavage of target transcripts via sequence complementarity with their targets—the small cleavage-inducing RNAs (sciRNAs) [46]. The majority of the sciRNAs were derived from sequences other than miRNAs or piRNAs and. interestingly, a significant fraction could be mapped to annotated genes [46]. The authors used exact sequence matching between sncRNAs and their potential targets and to identify sciRNAs, and they speculated that many other sciRNAs that function via imperfect match complementarity remain to be discovered [46]. In another striking example, the study by Ouvrard et al. found that sncRNAs could affect the function of human promoters separated by thousands of base pairs from the corresponding RNA-binding sites [60]. While the authors used exogenously supplied siRNAs, it is quite possible that some endogenous sncRNAs could function using a similar mechanism [60].

Interestingly, both studies by Cass et al. and Ouvrard et al. implicated members of the Argonaute (Ago) family of proteins [46], the key components of RISC complex that mediate miRNA and siRNA effects on their target genes in the RNAi pathway [61,62], in mediating the reported effects of sncRNAs on their targets. For example, Cass et al. showed that sciRNAs were bound by the Ago2 protein that is known to have RNA cleavage activity, and their function depended on Ago2, suggesting an intriguing possibility that novel sncRNAs could associate with known components of RNAi machinery and guide them to their targets [46]. Likewise, Ouvrard et al. showed that the long-distance effect of sncRNAs required both Ago1 and Ago2 [60]. These results are consistent with previous studies of Valen et al. and Burroughs et al. who found that TASRs, PASRs, and other sncRNAs could associate with different members of the Argonaute family [26,27]. Taken together, these results suggest that at least some novel sncRNAs could function by regulating other transcripts, via targeting of Ago or potentially other proteins, somewhat similar to the mechanisms of function of miRNAs or siRNAs [61,62]. Moreover, the strength of the phenotypic effects observed in our assay could, therefore, depend on the number and the type of transcripts regulated by an sncRNA.

It is also important to point out that any technique aimed at understanding the biological relevance of an RNA molecule, long or small, has limitations [48,63]. While highly suggestive of physiological relevance, our assay also had limitations; therefore, by itself, it does not provide a definitive proof that a large fraction of novel sncRNAs, even shared by multiple cell types, are functional. First, even if the overexpression construct is stably integrated in the genome, it is unlikely that every sncRNA will be successfully overexpressed. For example, as shown here, only 20/27 sncRNAs could be overexpressed in the transient transfection assay. The possible reasons could include, for example, limited concentrations of the factors necessary for sncRNA processing and/or stability in the cell. The failure to overexpress would lead to failure in detecting the biological relevance of the corresponding sncRNA in the assay. Second, we cannot be totally sure that which RNA species caused the phenotype. Since our assay is based on overexpression of a longer transcript containing the sncRNA of interest plus flanking regions, it is conceivable that a longer version of the sncRNA of interest, or even other sncRNAs, could also be produced from the same longer transcript and be responsible for the phenotype. It is also possible that the phenotype is mediated by the longer precursor transcript overexpressed in our system and not by the sncRNA. Third, the assay is performed in cultured cancer cells, which does not necessarily mean that these transcripts are functional in normal cells or important for the whole organism [48].

Nevertheless, our results argue that the unannotated sncRNA transcriptome could be a very large reservoir of unannotated functional sncRNAs that deserve further in-depth studies for at least two major reasons. First, sncRNAs can represent novel biomarkers. In fact, the potential of circulating miRNAs as biomarkers for various diseases has been well documented in multiple studies [64,65,66]. Strikingly, the study by Rounge et al. identified multiple circulating sncRNAs, for which the levels in human plasma were associated with age, sex, and other traits [67]. Interestingly, the trait-associated sncRNAs belonged to both annotated and unannotated classes of sncRNAs, including a class defined by the authors as “mRNA fragments” [67] that was similar to the exon-overlapping sncRNAs defined in this study. These results suggested that multiple different classes of sncRNAs have potential as biomarkers.

Second, novel functional sncRNAs could greatly enhance our understanding of regulatory processes happening in human cells, as illustrated by the aforementioned studies. In addition to discovering novel regulatory mechanisms, such studies can shed further light on the complexities of the genomic architecture, as illustrated by the *SRA1* (steroid receptor RNA activator 1) locus. *SRA1* encodes different functional long protein-coding and noncoding transcripts [68], as well as a novel miRNA generated from an intron using a noncanonical processing pathway [69]. Interestingly, our results suggest that physiologically relevant sncRNA species could be derived from exons of protein-coding genes, including UTR or CDS regions. This finding is consistent with a previous concept that a single genomic base pair could be part of different types of functional genomic elements [70] and, as exemplified by the *SRA1* locus, further underscores the complexity of the information encoded in the human genome.

## 4. Materials and Methods

### 4.1. Biological Material

Human chronic myelogenous leukemia and embryonic kidney cell lines K562 and HEK293 were obtained from the Cell Bank of Chinese Academy of Sciences and National Infrastructure of Cell-line Resource, respectively. K562 and HEK293FT cells were respectively maintained in RPMI 1640 (ThermoFisher Scientific, Waltham, MA, USA) and DMEM (Sigma, Shanghai, China) supplemented with 10% fetal bovine serum (ThermoFisher Scientific, Waltham, MA, USA) and 1% pen/strep (ThermoFisher Scientific, Waltham, MA, USA) at 37 °C in 5% CO_2_.

### 4.2. K562 sncRNA Profiling

Total RNA was isolated as follows: 1–2 million K562 cells were resuspended in 1 mL of TRNzol Universal reagent (TIANGEN, Beijing, China) and incubated at room temperature for 5 min for complete lysis. Following the addition of 0.2 mL of chloroform, the sample was vortexed vigorously, incubated for 3 min at room temperature, and centrifuged at 12,000× *g* for 15 min at 4 °C. The RNA was precipitated from the aqueous phase by adding 5 μg of RNase-free glycogen and 0.5 mL of 100% isopropyl alcohol, followed by incubation at room temperature for 10 min and centrifugation at 12,000× *g* for 10 min at 4 °C. The pellet was washed with 75% ethanol, dried, resuspended in RNase-free water, and used for small RNA-seq library construction with NEB Next^®^ Multiplex Small RNA Library Prep Set for Illumina^®^ (NEB) following the manufacturer’s instructions. After library construction, PCR products corresponding to small RNAs in the range of 20–40 nt were purified by PAGE and used for small RNA-seq. The sRNA-seq was performed on the Illumina platform (Hiseq 2500) by Novogene Inc. (Beijing, China) using a single-end 50 base strategy on a scale of 10–25 million reads. Raw reads, in which >10% of bases were N or >50% of bases had low (≤5) quality scores, as well as reads containing sequence adapters or long homopolymeric tracks, were removed. The quality-filtered reads were then aligned to the GRCh37/hg19 version of the human genome using Bowtie2 (v2.2.9) with default settings. Only reads uniquely mapping to the genome were kept.

The RPKM value was calculated for each unique alignment defined by unique 5′ and 3′ coordinates and the strand. The aligned reads that overlapped on the same strand were merged by BEDtools (v2.25.0); for each cluster, a representative sncRNA with unique 5′/3′ coordinates and the maximum RPKM was selected for the downstream analysis. To generate unannotated sncRNAs, prior to selection of representative sncRNAs, the merged clusters were first filtered to remove those that overlapped the following: (1) the RNA family of repeats as defined by the Repeat Masker, (2) annotated pre-miRNAs as defined by miRBase [33] (https://www.mirbase.org/, accessed on 1 July 2020), and (3) snoRNAs as defined by the sno/miRNA Track from the UCSC Genome Browser (http://genome.ucsc.edu/cgi-bin/hgTrackUi?hgsid=1528387543_JvQqnpmvlzYwNvlViZII2NvpzabA&db=hg19&c=chr2&g=wgRna, accessed on 11 August 2022). The clusters overlapping with the RNA family of repeats on either strand were removed; however, clusters overlapping with mi- or snoRNAs only on the same strand were removed. The K562 ENCODE small RNA-seq data were downloaded from the UCSC Genome Browser (http://www.genome.ucsc.edu/cgi-bin/hgFileUi?db=hg19&g=wgEncodeCshlShortRnaSeq, accessed on 11 August 2022).

The odds ratios *OR* of enrichment of the overlap of unannotated sncRNAs with a genomic element *i* relative to the random chance, as shown in Figure 2 and Supplementary Table S1, were calculated as follows:

$$OR_{i}=\frac{LM_{i}/T_{i}}{L_{i}/LG},$$

where *LM_i_* is the length of sncRNAs overlapping the genomic element *i*, *T_i_* is the total length of sncRNAs mapping to genome, *L_i_* is the total length of the genomic element *i*, and *LG* is the total length of the genome. Note that the total length of RNA family of repeats was removed from *L_i_* and *LG*.

The odds ratios *OR* of the enrichment of miRBase miRNAs relative to all sncRNAs in different phenotypic categories, as shown Figure 6e and Supplementary Table S5, were calculated as

$$OR_{i}=\frac{n_{i}/T_{i}}{37/404},$$

where *n_i_* and *T_i_* are respectively the numbers of positive miRBase miRNAs and total number of positive sncRNAs in each phenotypic category.

The odds ratios *OR* (Figure 6e and Supplementary Table S5) of the enrichment of unannotated sncRNAs in different genomic elements and in different phenotypic categories were calculated as

$$OR_{i}=\frac{n_{i}/T_{i}}{N_{i}/367},$$

where *n_i_* and *N_i_* are respectively the numbers of positive unannotated sncRNAs in each phenotypic category and all unannotated sncRNAs that correspond to the genomic element *i*, and *T_i_* is the total number of positive unannotated sncRNAs in each phenotypic category.

### 4.3. Validation of sncRNA Overexpression Using Transient Transfection

The genomic regions containing each of the 34 sncRNA plus upstream and downstream 200 bp were separately amplified using PCR from K562 genomic DNA and cloned into the lentiviral vector essentially as described below (Generation of sncRNA overexpression library) with the exception that the individual PCR products were not mixed for cloning. Instead, plasmids corresponding to overexpression vector containing each sncRNAs were prepared separately and mixed equally; then, 2.5 μg of the plasmid pool was used to transiently transfect one million HEK293 cells using Epfect Transfection Reagent (Syngentech, Beijing, China). After 8 h, the transfection medium was replaced with the fresh culture medium with or without Dox, and cells were cultured for additional 24 h. In parallel, cells transfected with an “empty” vector, without the sncRNA-containing regions, were used as a “blank” control and were grown without Dox (Macklin Inc, 24390-14-5, Steward, IL). Each transfection experiment was performed in parallel on two independent batches of cells. Transfected cells were then selected using the MoFlo Astrios EQ flow cell sorter (Beckman Coulter, Brea, CA, USA) using GFP expression. Total RNA was then isolated from the selected cells and subjected to sncRNA profiling using RNA-seq as described above.

### 4.4. Generation of sncRNA Overexpression Library

The genomic regions containing each 404 sncRNA plus upstream and downstream 200 bp were separately amplified using PCR from K562 genomic DNA with primers containing BsaI restriction sites. PCR amplification was performed using a high-fidelity Taq polymerase Phanta Max Super-Fidelity DNA Polymerase (Vazyme, Nanjing, China). After PCR amplification, all purified PCR products were combined, and 1 μg of the mixture was digested with 25 U BsaI (New England Biolabs, Ipswich, MA) at 37 °C for 12 h, followed by 20 min of incubation at 65 °C to inactivate the enzyme and purified with 0.8 volumes of VAHTS beads (Vazyme, Nanjing, China). The digested DNA (150 ng) was then ligated with 40 ng of BsaI-digested lentivirus vector pHS-AVC-LW1034 (SyngenTech, Beijing, China) in 20 μL using 600 U of T4 DNA ligase (New England Biolabs) at 16 °C overnight, followed by enzyme inactivation at 65 °C for 10 min. Four different ligation mixtures were set up in parallel, combined, and used directly to transform Stbl2 *E. coli* competent cells (Shanghai Weidi Biotechnology, Shanghai, China) using heat shock. The bacterial colonies containing the library plasmids were counted, and a sufficient number of them to ensure >100× coverage of each sequence were scraped from the agar plates directly and used for plasmid isolation using PureLinkTM HiPure Plasmid Filter Midiprep Kit (Invitrogen, Waltham, MA, USA) following the manufacturer’s protocol. Twenty bacterial colonies were selected randomly for Sanger sequencing check, with all of them containing different expected sequences.

The following protocol was outsourced to SyngenTech (Beijing, China): lentivirus particles were produced by transfecting the HEK 293FT packaging cell line with 2.5 μg of the library plasmid mixture. The resulting lentiviral library was then used to transfect the K562 cell line at an infection rate of 24.25% to favor single integration events. Two million stably transfected K562 cells containing the sncRNAs overexpression sequences (TRE-sRNA-K562) were selected by flow cytometry (BD CytoFLEX, Brea, CA, USA) using GFP fluorescence and expanded.

### 4.5. Detection of sncRNAs Affecting Cell Growth and Survival

One million TRE-sRNA-K562 cells (>2000× cells/sncRNA) were cultured in 10 mL of medium with or without 1 μg/mL Dox in a T25 flask with three independent biological replicates performed for each +Dox or −Dox treatment. The medium with or without Dox was changed every 2 days. After 16 and 32 days, aliquots of two million cells were used for genomic DNA isolation, while the remaining cells were allowed to continue in culture until day 60. Cells immediately prior to the culture were also used for DNA isolation, and this sample is referred to as “day 0”. Genomic DNA was isolated using TIANamp Genomic DNA Kit (Tiangen, Beijing, China).

For each sample, the sncRNA overexpression regions were amplified in five parallel PCR reactions as follows: each reaction contained 1 μg of genomic DNA, 1× Taq buffer, 0.2 mM of each dNTP (Takara, Beijing, China), 0.5 μM of each P5-Lenti-forward primer (CTACACGACGCTCTTCCGATCTAGCAGAGCTCGTTTAGTGAACCG) and P7-Lenti-reverse primer (GTTCAGACGTGTGCTCTTCCGATCTATGTGCGCTCTGCCCACTGA), and 2.5 U of Taq DNA polymerase (Tiangen, Beijing, China) in a total volume of 50 μL. PCR conditions were as follows: initial denaturation at 94 °C for 3 min; 10 cycles of denaturation at 94 °C for 30 s, annealing at 55 °C for 30 s, and extension at 72 °C for 1 min; further extension at 72 °C for another 10 min. Then, 2 μL of the first-round PCR product was used for the second-round amplification with 1× Taq buffer, 0.2 mM of each dNTP, 0.5 μM of each Illumina-P5 primer (AATGATACGGCGACCACCGAGATCTACACTCTTTCCCTACACGACGCTCTTCCGATCT) and Illumina-P7 primer (CAAGCAGAAGACGGCATACGAGATCGTGATGTGACTGGAGTTCAGACGTGTGCTCTTCCGATCT), and 1 U of Taq DNA polymerase in 20 μL. The PCR conditions were same as in the first-round PCR, except that 20 cycles of amplification were used. Each PCR reaction was purified with 0.8 volumes of VAHTS DNA Clean Beads, and the five PCR reactions for the same sample were pooled and subjected to NGS sequencing. The latter was performed on Hiseq X Ten platform using the paired-end 150 bp (PE150) strategy on a 2 Gb scale and outsourced to Novogene Corporation (Beijing, China).

Raw read pairs in which at least one read contained >10% of N bases and/or >50% of bases with low (≤5) quality scores were removed. The quality-filtered paired-end sequences were further filtered for the presence of expected sequence AGCAGAGCTCGTTTAGTGAACCGTCAGATCGCCTGGAGATTTCGTTAATTAAACCG at the beginning of read 1 and ATGTGCGCTCTGCCCACTGACGGGCACCGGAGCCAAGCTAGCTTTTGGTAGATCAGTTATAAAA at the beginning of read 2; then, both of the expected sequences tags were removed, and the read pairs were aligned to the human reference genome (GRCh37/hg19) using Bowtie 2 [71]. Aligned reads were assigned to each sncRNA region and used to generate counts for each region in each library. One NGS library was prepared for each biological replicate of +Dox or −Dox sample at the 16, 32, and 60 day timepoints. Two NGS libraries (two technical replicates) were generated for the day 0 samples. The read count for each sncRNA region in each sample was first normalized to the sequencing depth of the corresponding NGS library to generate the normalized read count (*NRC*), as shown below, where *RC* denotes the read counts for the sncRNA region *i* in library *j*, and *TRC* is the total read count in library *j*.

$$NRC_{i,j}=\frac{RC_{i,j}\times 10^{6}}{TRC_{j}}.$$

To define sncRNAs depleted or enriched in the +Dox compared to the −Dox samples, we used three metrics. First, the *NRC* of such sncRNAs had to be lower or higher at each timepoint in the +Dox samples compared to the −Dox ones. To assess this, we calculated adjusted fold change (*adj*.*FC*) for each sncRNA region *i* at each timepoint (16, 32, or 60 days) *t*, as shown below, where *avgNRC* referrers to the average NRC calculated for the sncRNA region across the three biological replicates of +Dox or −Dox samples at the corresponding timepoint.

$$adj.FC_{i,t}=\frac{avgNRC{\left(+Dox\right)}_{i,t}}{avgNRC{\left(-Dox\right)}_{i,t}+NRC{\left(+Dox\right)}_{i,t}}.$$

Second, we calculated the statistical significance of depletion or enrichment for each sncRNA sample in response to Dox using a one-sided Student’s paired *t*-test for each timepoint. The *p*-values were then adjusted for multiple comparisons with the Benjamini–Hochberg method in the R environment. Third, we calculated the fold change vs. the 0 day sample (*FC*0) for each sncRNA region *i* at each timepoint (16, 32, or 60 days) *t*, as shown below, using the average *NCR* of +Dox and −Dox samples compared to that of the *NCR* at the 0 day timepoint.

$$FC0_{i,t}=\frac{avgNCR\;{\left(+Dox\;and-Dox\right)}_{i,t}}{avgNCR_{i,0}}.$$

To define depleted sncRNAs that were detrimental to cell growth and survival, we used the following conditions: (1) *adj*.*FC* < 0.5 at each timepoint; (2) sncRNAs depleted at 60 days had to have adjusted *p*-values <0.05 at each timepoint; (3) sncRNAs depleted at 32 days had to have adjusted *p*-values <0.05 at both 16 and 32 day timepoints and *FC*0 < 0.2 at the 60 day timepoint; (4) sncRNAs depleted at 16 days had to have adjusted *p*-values <0.05 at that timepoint and *FC*0 < 0.2 at both 32 and 60 day timepoints. The rationale for using the *FC*0 < 0.2 criterion was based on the observation that, due to the leaky expression, overexpression of sncRNAs with a strong phenotypic effect could eventually become toxic to cells even without Dox, resulting in very few or no cells harboring such sncRNAs in +Dox or −Dox samples at later timepoints. Such loss of cells would preclude accurate *p*-value calculations at the later timepoints.

To define enriched sncRNAs that could promote cell growth and survival, we used the following conditions: (1) *adj*.*FC* > 0.5 at each timepoint; (2) sncRNAs enriched at 16 days had to have adjusted *p*-values < 0.05 at each time point; (3) sncRNAs enriched at 32 days had to have adjusted *p*-values < 0.05 at both 32 and 60 day timepoints and *FC*0 >1 at the 16 day timepoint; (4) sncRNAs enriched at 60 days had to have adjusted *p*-values < 0.05 at that timepoint and *FC*0 > 1 at 16, 32, and 60 day timepoints. The rationale for using the *FC*0 > 1 criterion was based on a possibility that the effect of an sncRNA may be weak and would show itself only at the later timepoints. However, no sncRNAs that could promote cell growth or survival were identified in this study.

## Data Availability

The NGS data were submitted to GEO with accession number GSE221958.

## Supplementary Materials

The following supporting information can be downloaded at https://www.mdpi.com/article/10.3390/ijms24044163/s1.

## References

[1] Kapranov P., Cawley S.E., Drenkow J., Bekiranov S., Strausberg R.L., Fodor S.P., Gingeras T.R. (2002). Large-scale transcriptional activity in chromosomes 21 and 22. Science.

[2] Okazaki Y., Furuno M., Kasukawa T., Adachi J., Bono H., Kondo S., Nikaido I., Osato N., Saito R., Suzuki H. (2002). Analysis of the mouse transcriptome based on functional annotation of 60,770 full-length cDNAs. Nature.

[3] Rinn J.L., Euskirchen G., Bertone P., Martone R., Luscombe N.M., Hartman S., Harrison P.M., Nelson F.K., Miller P., Gerstein M. (2003). The transcriptional activity of human Chromosome 22. Genes Dev..

[4] Bertone P., Stolc V., Royce T.E., Rozowsky J.S., Urban A.E., Zhu X., Rinn J.L., Tongprasit W., Samanta M., Weissman S. (2004). Global identification of human transcribed sequences with genome tiling arrays. Science.

[5] Cheng J., Kapranov P., Drenkow J., Dike S., Brubaker S., Patel S., Long J., Stern D., Tammana H., Helt G. (2005). Transcriptional maps of 10 human chromosomes at 5-nucleotide resolution. Science.

[6] Carninci P., Kasukawa T., Katayama S., Gough J., Frith M.C., Maeda N., Oyama R., Ravasi T., Lenhard B., Wells C. (2005). The transcriptional landscape of the mammalian genome. Science.

[7] Kapranov P., Cheng J., Dike S., Nix D.A., Duttagupta R., Willingham A.T., Stadler P.F., Hertel J., Hackermuller J., Hofacker I.L. (2007). RNA maps reveal new RNA classes and a possible function for pervasive transcription. Science.

[8] Djebali S., Davis C.A., Merkel A., Dobin A., Lassmann T., Mortazavi A., Tanzer A., Lagarde J., Lin W., Schlesinger F. (2012). Landscape of transcription in human cells. Nature.

[9] Mattick J.S., Amaral P.P., Carninci P., Carpenter S., Chang H.Y., Chen L.L., Chen R., Dean C., Dinger M.E., Fitzgerald K.A. (2023). Long non-coding RNAs: Definitions, functions, challenges and recommendations. Nat. Rev. Mol. Cell Biol..

[10] Clark M.B., Choudhary A., Smith M.A., Taft R.J., Mattick J.S. (2013). The dark matter rises: The expanding world of regulatory RNAs. Essays Biochem..

[11] Kapranov P., St Laurent G. (2012). Dark Matter RNA: Existence, Function, and Controversy. Front. Genet..

[12] Kapranov P., St Laurent G., Raz T., Ozsolak F., Reynolds C.P., Sorensen P.H., Reaman G., Milos P., Arceci R.J., Thompson J.F. (2010). The majority of total nuclear-encoded non-ribosomal RNA in a human cell is ‘dark matter’ un-annotated RNA. BMC Biol..

[13] Mattick J.S. (2003). Challenging the dogma: The hidden layer of non-protein-coding RNAs in complex organisms. Bioessays.

[14] Mattick J.S. (2007). A new paradigm for developmental biology. J. Exp. Biol..

[15] St Laurent G., Wahlestedt C., Kapranov P. (2015). The Landscape of long noncoding RNA classification. Trends Genet..

[16] Ponting C.P., Haerty W. (2022). Genome-Wide Analysis of Human Long Noncoding RNAs: A Provocative Review. Annu. Rev. Genom. Hum. Genet..

[17] Morris K.V., Mattick J.S. (2014). The rise of regulatory RNA. Nat. Rev. Genet..

[18] Kung J.T., Colognori D., Lee J.T. (2013). Long noncoding RNAs: Past, present, and future. Genetics.

[19] Taft R.J., Glazov E.A., Cloonan N., Simons C., Stephen S., Faulkner G.J., Lassmann T., Forrest A.R., Grimmond S.M., Schroder K. (2009). Tiny RNAs associated with transcription start sites in animals. Nat. Genet..

[20] Taft R.J., Simons C., Nahkuri S., Oey H., Korbie D.J., Mercer T.R., Holst J., Ritchie W., Wong J.J., Rasko J.E. (2010). Nuclear-localized tiny RNAs are associated with transcription initiation and splice sites in metazoans. Nat. Struct. Mol. Biol..

[21] Seila A.C., Calabrese J.M., Levine S.S., Yeo G.W., Rahl P.B., Flynn R.A., Young R.A., Sharp P.A. (2008). Divergent transcription from active promoters. Science.

[22] Kapranov P., Ozsolak F., Kim S.W., Foissac S., Lipson D., Hart C., Roels S., Borel C., Antonarakis S.E., Monaghan A.P. (2010). New class of gene-termini-associated human RNAs suggests a novel RNA copying mechanism. Nature.

[23] Laudadio I., Formichetti S., Gioiosa S., Klironomos F., Rajewsky N., Macino G., Carissimi C., Fulci V. (2018). Characterization of Transcription Termination-Associated RNAs: New Insights into their Biogenesis, Tailing, and Expression in Primary Tumors. Int. J. Genom..

[24] Ma X., Han N., Shao C., Meng Y. (2017). Transcriptome-Wide Discovery of PASRs (Promoter-Associated Small RNAs) and TASRs (Terminus-Associated Small RNAs) in Arabidopsis thaliana. PLoS ONE.

[25] Leng X.M., Diao L.T., Li B., Bi Y.Z., Chen C.J., Zhou H., Qu L.H. (2014). The ribosomal protein rpl26 promoter is required for its 3’ sense terminus ncRNA transcription in Schizosaccharomyces pombe, implicating a new transcriptional mechanism for ncRNAs. Biochem. Biophys. Res. Commun..

[26] Valen E., Preker P., Andersen P.R., Zhao X., Chen Y., Ender C., Dueck A., Meister G., Sandelin A., Jensen T.H. (2011). Biogenic mechanisms and utilization of small RNAs derived from human protein-coding genes. Nat. Struct. Mol. Biol..

[27] Burroughs A.M., Ando Y., de Hoon M.J., Tomaru Y., Suzuki H., Hayashizaki Y., Daub C.O. (2011). Deep-sequencing of human Argonaute-associated small RNAs provides insight into miRNA sorting and reveals Argonaute association with RNA fragments of diverse origin. RNA Biol..

[28] Ghildiyal M., Zamore P.D. (2009). Small silencing RNAs: An expanding universe. Nat. Rev. Genet..

[29] Ha M., Kim V.N. (2014). Regulation of microRNA biogenesis. Nat. Rev. Mol. Cell Biol..

[30] Desvignes T., Batzel P., Berezikov E., Eilbeck K., Eppig J.T., McAndrews M.S., Singer A., Postlethwait J.H. (2015). miRNA Nomenclature: A View Incorporating Genetic Origins, Biosynthetic Pathways, and Sequence Variants. Trends Genet..

[31] Ozata D.M., Gainetdinov I., Zoch A., O’Carroll D., Zamore P.D. (2019). PIWI-interacting RNAs: Small RNAs with big functions. Nat. Rev. Genet..

[32] Kiss T. (2002). Small nucleolar RNAs: An abundant group of noncoding RNAs with diverse cellular functions. Cell.

[33] Kozomara A., Birgaoanu M., Griffiths-Jones S. (2019). miRBase: From microRNA sequences to function. Nucleic Acids Res..

[34] Leung Y.Y., Kuksa P.P., Amlie-Wolf A., Valladares O., Ungar L.H., Kannan S., Gregory B.D., Wang L.S. (2016). DASHR: Database of small human noncoding RNAs. Nucleic Acids Res..

[35] Wang J., Zhang P., Lu Y., Li Y., Zheng Y., Kan Y., Chen R., He S. (2019). piRBase: A comprehensive database of piRNA sequences. Nucleic Acids Res..

[36] Bouchard-Bourelle P., Desjardins-Henri C., Mathurin-St-Pierre D., Deschamps-Francoeur G., Fafard-Couture E., Garant J.M., Elela S.A., Scott M.S. (2020). snoDB: An interactive database of human snoRNA sequences, abundance and interactions. Nucleic Acids Res..

[37] Wei W., Ba Z., Gao M., Wu Y., Ma Y., Amiard S., White C.I., Rendtlew Danielsen J.M., Yang Y.G., Qi Y. (2012). A role for small RNAs in DNA double-strand break repair. Cell.

[38] Francia S., Michelini F., Saxena A., Tang D., de Hoon M., Anelli V., Mione M., Carninci P., d’Adda di Fagagna F. (2012). Site-specific DICER and DROSHA RNA products control the DNA-damage response. Nature.

[39] Gao M., Wei W., Li M.M., Wu Y.S., Ba Z., Jin K.X., Li M.M., Liao Y.Q., Adhikari S., Chong Z. (2014). Ago2 facilitates Rad51 recruitment and DNA double-strand break repair by homologous recombination. Cell Res..

[40] Tuck A.C., Tollervey D. (2011). RNA in pieces. Trends Genet..

[41] Pederson T. (2010). Regulatory RNAs derived from transfer RNA?. RNA.

[42] Malka Y., Alkan F., Ju S., Korner P.R., Pataskar A., Shulman E., Loayza-Puch F., Champagne J., Wenzel C., Faller W.J. (2022). Alternative cleavage and polyadenylation generates downstream uncapped RNA isoforms with translation potential. Mol. Cell.

[43] Fejes-Toth K., Sotirova V., Sachidanandam R., Assaf G., Hannon G.J., Kapranov P., Foissac S., Willingham A.T., Duttagupta R., Dumais E. (2009). Post-transcriptional processing generates a diversity of 5’-modified long and short RNAs. Nature.

[44] Mercer T.R., Dinger M.E., Bracken C.P., Kolle G., Szubert J.M., Korbie D.J., Askarian-Amiri M.E., Gardiner B.B., Goodall G.J., Grimmond S.M. (2010). Regulated post-transcriptional RNA cleavage diversifies the eukaryotic transcriptome. Genome Res..

[45] Wilusz J.E., Freier S.M., Spector D.L. (2008). 3’ end processing of a long nuclear-retained noncoding RNA yields a tRNA-like cytoplasmic RNA. Cell.

[46] Cass A.A., Bahn J.H., Lee J.H., Greer C., Lin X., Kim Y., Hsiao Y.H., Xiao X. (2016). Global analyses of endonucleolytic cleavage in mammals reveal expanded repertoires of cleavage-inducing small RNAs and their targets. Nucleic Acids Res..

[47] Bartel D.P. (2009). MicroRNAs: Target recognition and regulatory functions. Cell.

[48] Gao F., Cai Y., Kapranov P., Xu D. (2020). Reverse-genetics studies of lncRNAs—What we have learnt and paths forward. Genome Biol..

[49] Jin H.Y., Gonzalez-Martin A., Miletic A.V., Lai M., Knight S., Sabouri-Ghomi M., Head S.R., Macauley M.S., Rickert R.C., Xiao C. (2015). Transfection of microRNA Mimics Should Be Used with Caution. Front. Genet..

[50] Treiber T., Treiber N., Meister G. (2019). Regulation of microRNA biogenesis and its crosstalk with other cellular pathways. Nat. Rev. Mol. Cell Biol..

[51] Chiang H.R., Schoenfeld L.W., Ruby J.G., Auyeung V.C., Spies N., Baek D., Johnston W.K., Russ C., Luo S., Babiarz J.E. (2010). Mammalian microRNAs: Experimental evaluation of novel and previously annotated genes. Genes Dev..

[52] Chen C.Z., Li L., Lodish H.F., Bartel D.P. (2004). MicroRNAs modulate hematopoietic lineage differentiation. Science.

[53] Pham D.H., Moretti P.A., Goodall G.J., Pitson S.M. (2008). Attenuation of leakiness in doxycycline-inducible expression via incorporation of 3’ AU-rich mRNA destabilizing elements. Biotechniques.

[54] Hosoya O., Chung M., Ansai S., Takeuchi H., Miyaji M. (2021). A modified Tet-ON system minimizing leaky expression for cell-type specific gene induction in medaka fish. Dev. Growth Differ..

[55] Costello A., Lao N.T., Gallagher C., Capella Roca B., Julius L.A.N., Suda S., Ducree J., King D., Wagner R., Barron N. (2019). Leaky Expression of the TET-On System Hinders Control of Endogenous miRNA Abundance. Biotechnol. J..

[56] Nechaev S., Fargo D.C., dos Santos G., Liu L., Gao Y., Adelman K. (2010). Global analysis of short RNAs reveals widespread promoter-proximal stalling and arrest of Pol II in Drosophila. Science.

[57] Xu D., Cai Y., Tang L., Han X., Gao F., Cao H., Qi F., Kapranov P. (2020). A CRISPR/Cas13-based approach demonstrates biological relevance of vlinc class of long non-coding RNAs in anticancer drug response. Sci. Rep..

[58] Isakova A., Neff N., Quake S.R. (2021). Single-cell quantification of a broad RNA spectrum reveals unique noncoding patterns associated with cell types and states. Proc. Natl. Acad. Sci. USA.

[59] Liu S.J., Nowakowski T.J., Pollen A.A., Lui J.H., Horlbeck M.A., Attenello F.J., He D., Weissman J.S., Kriegstein A.R., Diaz A.A. (2016). Single-cell analysis of long non-coding RNAs in the developing human neocortex. Genome Biol..

[60] Ouvrard J., Muniz L., Nicolas E., Trouche D. (2022). Small Interfering RNAs Targeting a Chromatin-Associated RNA Induce Its Transcriptional Silencing in Human Cells. Mol. Cell. Biol..

[61] Hutvagner G., Simard M.J. (2008). Argonaute proteins: Key players in RNA silencing. Nat. Rev. Mol. Cell Biol..

[62] Muller M., Fazi F., Ciaudo C. (2019). Argonaute Proteins: From Structure to Function in Development and Pathological Cell Fate Determination. Front. Cell Dev. Biol..

[63] Cao H., Wahlestedt C., Kapranov P. (2018). Strategies to Annotate and Characterize Long Noncoding RNAs: Advantages and Pitfalls. Trends Genet..

[64] Kaneto C.M., Nascimento J.S., Prado M., Mendonca L.S.O. (2019). Circulating miRNAs as biomarkers in cardiovascular diseases. Eur. Rev. Med. Pharmacol. Sci..

[65] Sohel M.M.H. (2020). Circulating microRNAs as biomarkers in cancer diagnosis. Life Sci..

[66] Sabre L., Punga T., Punga A.R. (2020). Circulating miRNAs as Potential Biomarkers in Myasthenia Gravis: Tools for Personalized Medicine. Front. Immunol..

[67] Rounge T.B., Umu S.U., Keller A., Meese E., Ursin G., Tretli S., Lyle R., Langseth H. (2018). Circulating small non-coding RNAs associated with age, sex, smoking, body mass and physical activity. Sci. Rep..

[68] Hube F., Velasco G., Rollin J., Furling D., Francastel C. (2011). Steroid receptor RNA activator protein binds to and counteracts SRA RNA-mediated activation of MyoD and muscle differentiation. Nucleic Acids Res..

[69] Hube F., Ulveling D., Sureau A., Forveille S., Francastel C. (2017). Short intron-derived ncRNAs. Nucleic Acids Res..

[70] Kapranov P., Willingham A.T., Gingeras T.R. (2007). Genome-wide transcription and the implications for genomic organization. Nat. Rev. Genet..

[71] Langmead B., Salzberg S.L. (2012). Fast gapped-read alignment with Bowtie 2. Nat. Methods.

