# Supplementary material for: Evidence for Existence of Multiple Functional Human Small RNAs Derived from Transcripts of Protein-Coding Genes
Source: Int J Mol Sci. 2023 Feb 19;24(4):4163. doi: 10.3390/ijms24044163 (PMC9959880; doi:10.3390/ijms24044163)
Supplement: Supplementary file 1 [file ijms-24-04163-s001.zip › Gao_etal_Supplementary figure.pdf]

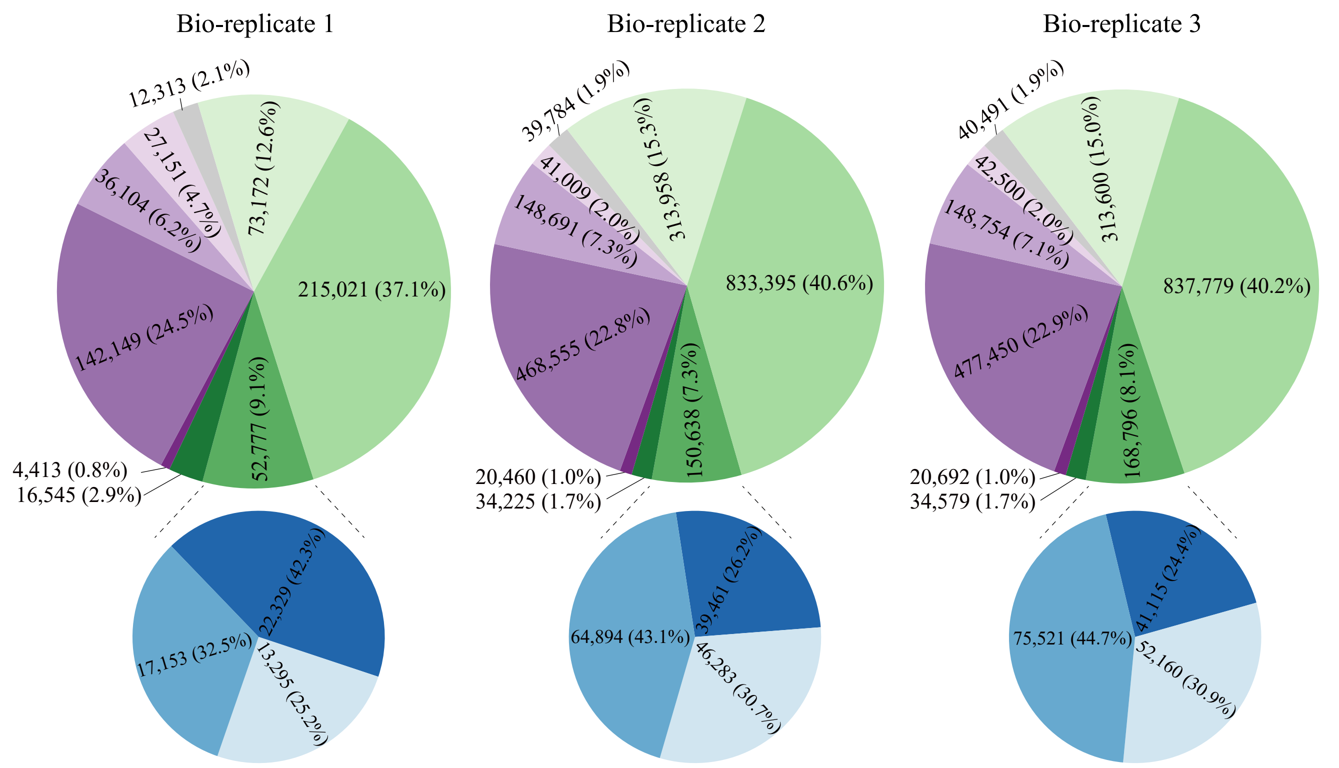


**Supplementary Figure S1. Very similar genomic profiles among the 3 biological replicates of K562 small RNA-seq data.** Distribution of numbers and fractions of uniquely mapping reads among the different genomic features for each biological replicate. Important, this analysis was done on all uniquely mapping reads, prior to merging shown in the Figure 1.
